# Review of disease-related complications and management in adult patients with thalassemia: A multi-center study in Thailand

**DOI:** 10.1371/journal.pone.0214148

**Published:** 2019-03-20

**Authors:** Suporn Chuncharunee, Nattiya Teawtrakul, Noppadol Siritanaratkul, Nonlawan Chueamuangphan

**Affiliations:** 1 Division of Hematology, Department of Internal Medicine, Faculty of Medicine, Ramathibodi Hospital, Mahidol University, Bangkok, Thailand; 2 Division of Hematology, Department of Internal Medicine, Faculty of Medicine, Srinagarind Hospital, Khon Kaen University, Khon Kaen, Thailand; 3 Division of Hematology, Department of Internal Medicine, Faculty of Medicine, Siriraj Hospital, Mahidol University, Bangkok, Thailand; 4 Hematology Unit, Chiang Rai Regional Hospital, Chiang Rai, Thailand; National Institutes of Health, National institute of Diabetes and Digestive and Kidney Diseases, UNITED STATES

## Abstract

Disease-related complications and management are different among patients with thalassemia. This study was aimed to review the prevalence, clinical risk factors for the complications and the management in patients with thalassemia in Thailand. A multicenter cross-sectional study was conducted in patients with thalassemia aged ≥ 18 years old. Thalassemia-related complications and management were reviewed. The clinical parameters significantly associated with the complications were analyzed by logistic regression methods. The prevalence of thalassemia-related complications was 100% in patients with transfusion-dependent thalassemia (TDT) and 58.8% in patients with non-transfusion-dependent thalassemia (NTDT). Advanced age was statistically associated with extramedullary hematopoiesis in both TDT and NTDT patients. Splenectomy was a significant risk factor for pulmonary hypertension in both groups of patients. Severe iron overload started earlier in patients with TDT than NTDT and was associated with diabetes mellitus (adjusted odds ratio (AOR) = 6.2, p-value = 0.02). Disease-related complications are more prevalent in patients with TDT than patients with NTDT. Splenectomy and advanced age were important risk factors for developing major complications in both groups. Early screening and management for specific disease-related complications should be considered in patients with thalassemia according to their clinical risk factors.

## Introduction

Thalassemia syndrome is the most common inherited disorder worldwide. The prevalence of thalassemia carriers is high among the populations in the Mediterranean, Eastern European and the Southeast Asian regions.[1] The severity of the disease depends on the degree of imbalance in the quantity of the globin chains. β-thalassemia is an example of this syndrome, caused by a defective quality and quantity of the beta globin chain production resulting in a decreased quantity of beta globin chains. The decrease in beta-globin chain production results in an excess of alpha globin chains; the more excessive alpha globin chains cause a more severe early apoptosis and ineffective erythropoiesis in the bone marrow. There is wide clinical diversity between the genotypes and phenotypes in patients with beta-thalassemia that is caused by several mechanisms e.g., the heterogeneity of mutations, secondary/tertiary modifiers, and environmental factors.[2] Evidence has shown several mechanisms that can reduce the excess of alpha globin chains resulting in improving the clinical phenotypes. For example, the coexistence of beta-thalassemia and alpha-thalassemia and an increase in Hb F production.[3,4] Presently, red blood cell transfusion requirements have been used to classify the patients with thalassemia into 2 major groups that include 1) transfusion-dependent thalassemia (TDT), and 2) non-transfusion-dependent thalassemia (NTDT). Transfusion-dependent thalassemia refers to the patients who require a regular blood transfusion for survival that includes patients with severe forms of thalassemia e.g., homozygous β^0^-thalassemia or hemoglobin E/β-thalassemia. Non-transfusion-dependent thalassemia refers to those patients who require an occasional red blood transfusion in certain circumstances e.g., surgery, pregnancy or infection. The patients with NTDT includes patients with a moderate severity of thalassemia e.g., hemoglobin H disease and some cases of hemoglobin E/β-thalassemia.[5]

Previous studies have demonstrated several disease-related complications in patients with thalassemia. The diversity of disease-related complications among patients with thalassemia is complicated by the wide spectrum of genotypes and clinical risk factors. Endocrine disorders and left ventricular disorders are more prevalent in patients with TDT.[6–10] Thrombosis, pulmonary hypertension, right ventricular disorders, and silent cerebral infarction are more common in patients with NTDT.[11–14] Moreover, a specific clinical risk factor, splenectomy, is a strong risk factor for pulmonary hypertension and thrombosis in patients with thalassemia.[15–18] Tailoring treatment based on patient specific risk factors should be the optimal management in patients with thalassemia in a country with limited resources. This multi-center study was aimed to review the disease-related complications and management in patients with TDT and NTDT in Thailand.

## Methods

A multicenter cross-sectional study was conducted in adult patients with thalassemia at Srinagarind Hospital (Khon Kaen University), Ramathibodi Hospital (Mahidol University), Siriraj Hospital (Mahidol University), and Chiang Rai Regional Hospital from September 30, 2015 to September 30, 2017. Eligible participants were patients with thalassemia aged ≥ 18 years old. All participants were evaluated for a history of disease-related complications, the red blood cell transfusion requirements, history of splenectomy, iron chelation therapy and concurrent medications. Laboratory data and investigations of risk factors identified in the literature for developing disease-related complications were collected. Further investigations into diagnosis and possible complications were performed if clinically indicated according to local guidelines.

### Disease-related complications that are possible sequelae of thalassemia syndromes include

1) Pulmonary hypertension defined as the presence of clinical suspicion of pulmonary hypertension and a right ventricular systolic pressure > 36 mmHg by trans-thoracic echocardiography.[19]
2) Heart failure that was defined as the presence or the history of signs and symptoms of heart failure according to the Framingham criteria.[20]
3) Diabetes mellitus was defined as the fasting plasma glucose ≥ 126 mg/dl.[21]
4) Extramedullary hematopoiesis was defined as the presence of clinical signs and symptoms or evidence of extramedullary hematopoiesis by ultrasonography, computed tomography scan (CT scan) or magnetic resonance imaging (MRI).
5) Gallstones were defined as the presence of gallstones in the gallbladder by ultrasonography.
6) Hypothyroidism was defined by the presence of elevation of serum TSH more than the upper limit and the level of free T4 that was lower than normal range.
7) Osteoporosis was defined as the presence of pathological fracture or the bone mineral density T-score < 2.5 SD. [22]
8) Thrombosis was defined by the presence of clinical signs and symptoms of thrombosis or the evidence of thrombosis by computed tomography angiogram (CT angiogram), computed tomography scan (CT scan), venography, angiography, doppler ultrasonography, or magnetic resonance imaging (MRI).
9) Infection was defined as the history of infections or the presence of clinical signs and symptoms of infections which were confirmed by the isolation of pathogens from blood, pus, stools, cerebrospinal fluid (CSF) or other body fluids.
10) Leg ulcers were defined by the presence of chronic venous ulcers on the legs.

#### Transfusion dependent thalassemia (TDT)

defined as a group of patients with thalassemia who require regular blood transfusions every 2–6 weeks for survival.

#### Non-transfusion dependent thalassemia (NTDT)

defined as a group of patients with thalassemia who do not require regular blood transfusions for survival, but may require an occasional blood transfusion. [1]

All participants gave written informed consent, and the research protocol was approved by the Human Research Ethics Review Boards of the Faculty of Medicine, Khon Kaen University, Mahidol University, and Chiang Rai Regional Hospital.

### Statistical analyses

Continuous variables were reported as means and standard deviations (SD) for normal distribution data and reported as median (minimum-maximum) if the data were not of a normal distribution. Categorical variables were reported as numbers and percentages. Odds ratios (OR) were used to demonstrate the associations between clinical risk factors and thalassemia-related complications in univariate logistic regressions. Multivariate logistic regressions included the significant variables in which the p-values were <0.2 in the univariate logistic regression or when the literature indicated significant risk factors. All statistical analyses were performed using STATA statistical software version 10 (StataCorp, College Station, TX). A p-value of less than 0.05 was considered statistically significant.

## Results and discussion

**Fig 1:**
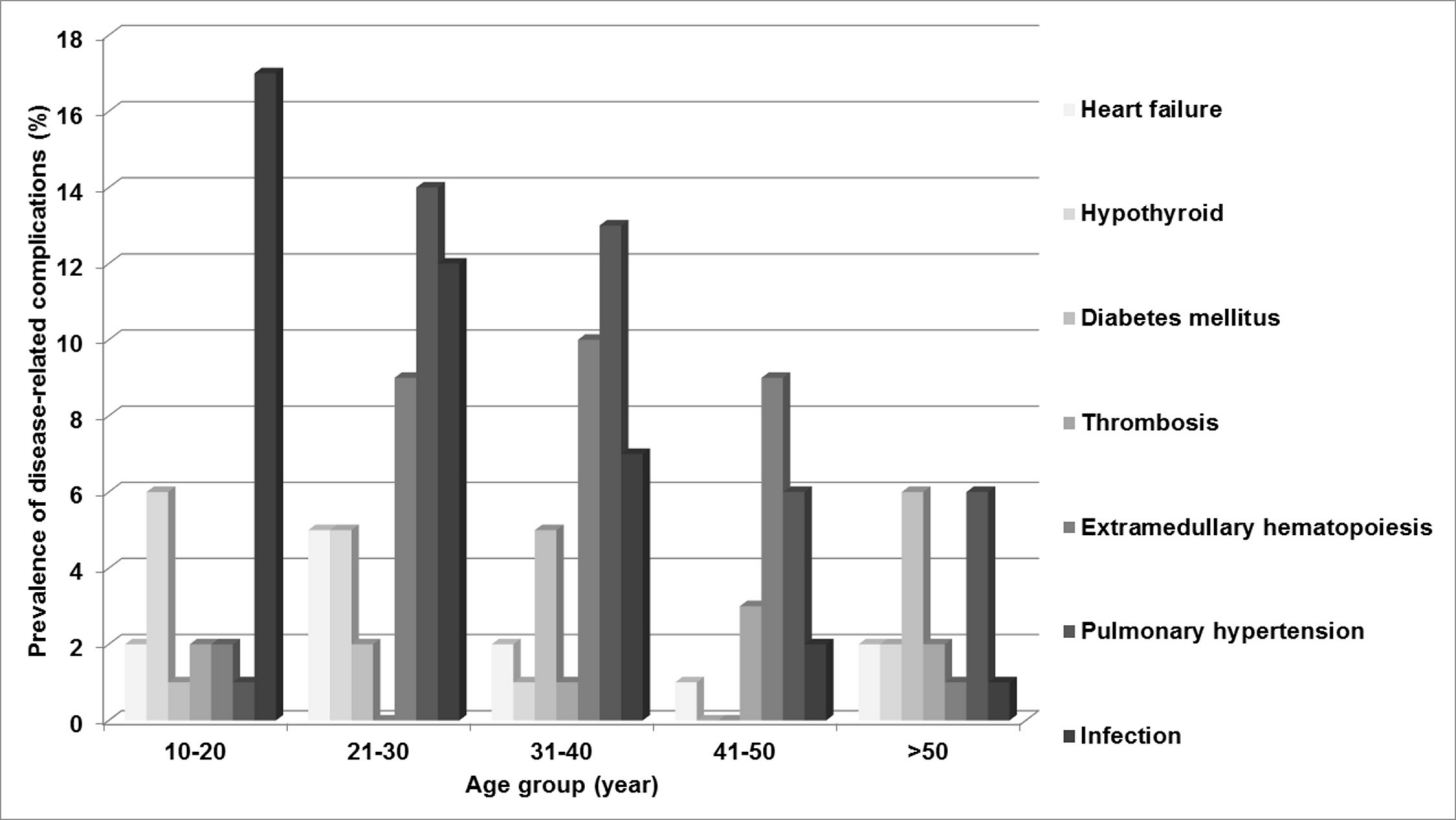
Prevalence of the disease-related complications by age group in the 433 patients with thalassemia.

**Fig 2:**
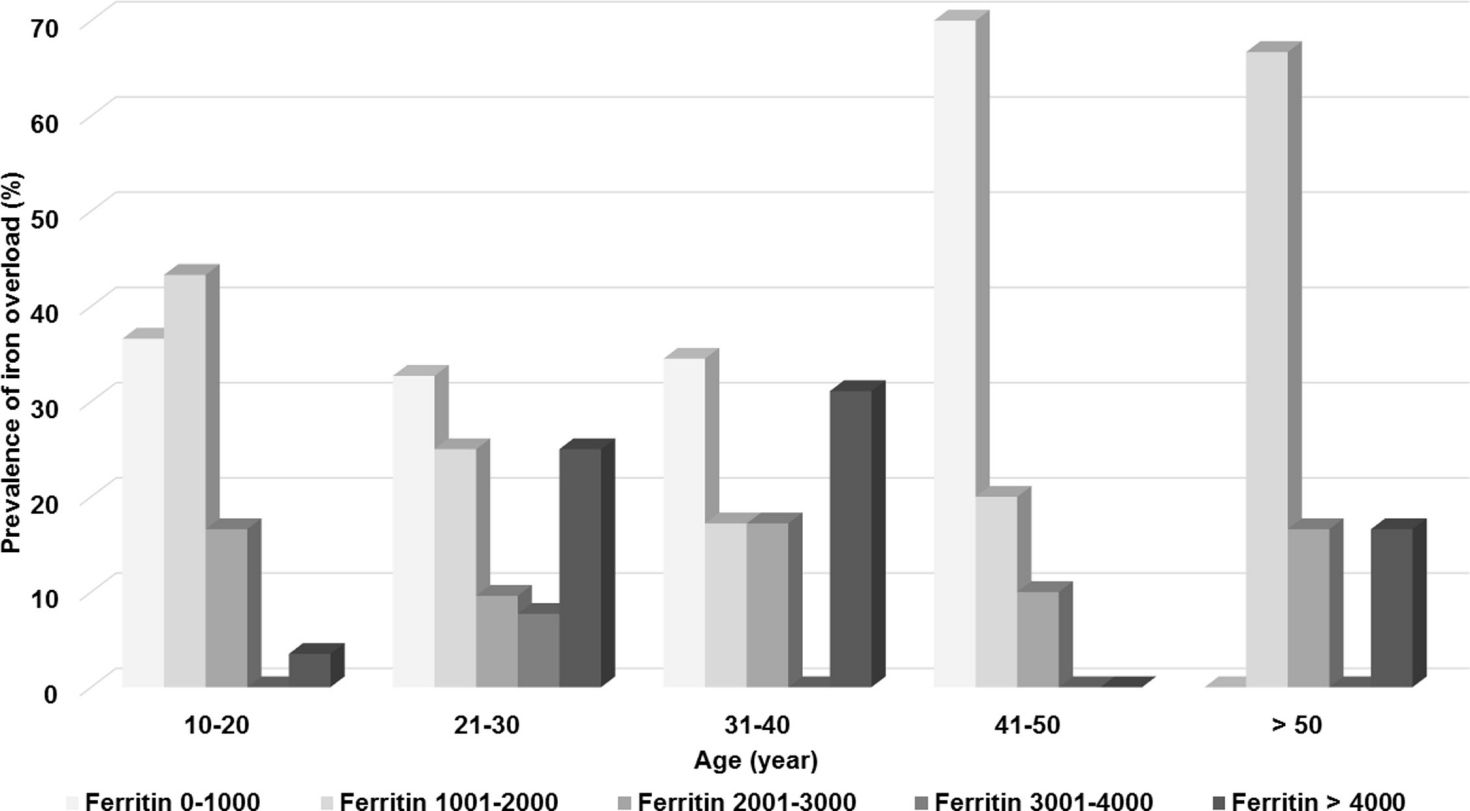
Prevalence of the severity of iron overload by age group in the 127 patients with transfusion-dependent thalassemia (TDT).

**Fig 3:**
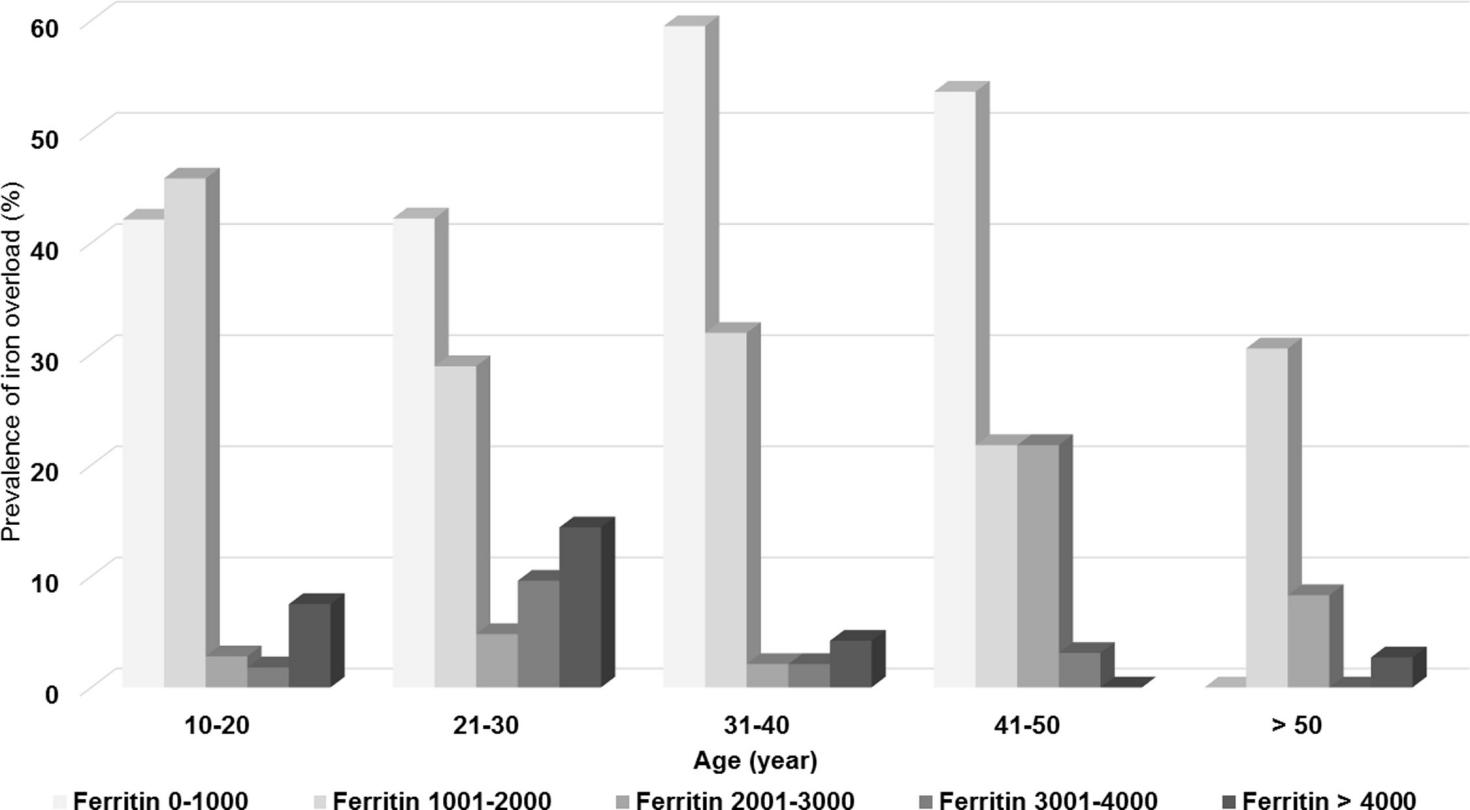
Prevalence of the severity of iron overload by age group in the 306 patients with non-transfusion-dependent thalassemia (NTDT).

A total of 433 patients (254 females, 179 males) were enrolled in the study. Non-transfusion-dependent thalassemia was more prevalent than transfusion-dependent-thalassemia (306 patients vs. 127 patients). The mean hemoglobins were 7.1 ± 1.3 g/dl in patients with TDT and 7.7 ± 1.2 g/dl in patients with NTDT. The mean serum ferritins were 2,250 ± 2,313 ng/ml in patients with TDT, and 1,483 ± 1,530 ng/ml in patients with NTDT. All of the patients with TDT had disease-related complications. Fewer than half of the patients with NTDT had no history of previous disease-related complications (126 patients, 41.2%). Baseline characteristics of the 433 patients with thalassemia are summarized in Table 1. The mean ages of patients were similar between the TDT and NTDT groups, 27.8 years vs. 29 years. Splenectomies were fewer in patients with TDT (56 patients, 48.4%) than those patients with NTDT (191 patients, 62.4%). Most of the splenectomized patients underwent the splenectomy surgery when they were young, and the aim of splenectomy was to decrease the transfusion and iron chelation requirement. Moreover, at that time of splenectomy, oral iron chelation did not exist. The most common disease-related complications in patients with TDT were gallstones followed by extramedullary hematopoiesis and pulmonary hypertension. In patients with NTDT, the most common disease-related complications were gallstones followed by infection and pulmonary hypertension. The most common iron chelation therapy was deferiprone followed by the combination of deferoxamine and deferiprone. Elevation of liver enzymes was the most common side effect of iron chelation in both groups of patients followed by gastrointestinal disturbances and arthralgia. Aspirin was the most common concomitant medication in the patients with TDT. Calcium supplements were the most common concomitant medications in the patients with NTDT. β-thalassemia/Hb E was the most common genotype in both groups. Fig 1 shows the prevalence of disease-related complications by age group. Most of the major disease-related complications included heart failure, pulmonary hypertension, extramedullary hematopoiesis, and endocrinopathies commonly found in the second and third decades of life. Infection was the only complication that was more prevalent in the first decade of life and the prevalence was slightly decreased in advanced age. Fig 2 shows the prevalence of the severity of iron overload by age group in the patients with TDT. Severe iron overload was found as early in the first decade of life with the peak prevalence in the second and third decades of life. Fig 3 shows the prevalence of the severity of iron overload by age group in the patients with NTDT. Severe iron overload started in the second decade of life with the peak prevalence in the third and fourth decades of life. The multivariate analysis of clinical risk factors for disease-related complications in patients with TDT is shown in Table 2. Age > 25 years was a significant clinical risk factor for pulmonary hypertension, infections, extramedullary hematopoiesis, hypogonadism, and gallstones. Post-splenectomy was a significant clinical risk factor for pulmonary hypertension and hypogonadism. Serum ferritin > 1,000 ng/ml was a statistically significantly risk factor for diabetes mellitus in patients with TDT (AOR = 6.2, 95% CI 1.2–30.8 and p-value = 0.02). The multivariate analysis of clinical risk factors for disease-related complications in patients with NTDT is shown in Table 3. Age > 25 years and post-splenectomy were significant clinical risk factors for pulmonary hypertension, heart failure, and extramedullary hematopoiesis. Serum ferritin > 1,000 ng/ml was a statistically significant risk factor for infections in patients with NTDT (AOR = 1.1, 95% CI 1.01–1.04 and p-value = 0.04).

**Table 1:** Baseline clinical characteristics of 433 patients with thalassemia.

Characteristics	TDT (n = 127)	NTDT (n = 306)
Mean age ± SD, years (at enrollment)	27.8 ± 11.4	29 ± 14.7
Mean hemoglobin ± SD, g/dL	7.1 ± 1.3	7.7 ± 1.2
Mean serum ferritin ± SD, ng/mL	2,250 ± 2,313	1,483 ± 1,530
Liver size ± SD, cm	3.2 ± 2.8	2.9 ± 3
Time after splenectomy	4.7 ± 13.6	1.9 ± 0.5
Gender, n (%)		
- Female	78 (61)	179 (57.5)
- Male	49 (39)	130 (42.5)
Splenectomy, n (%)		
- No	71 (55.9)	115 (37.6)
- Yes	56 (44.1)	191 (62.4)
Previous complications, n (%)		
- No	0 (0)	1261.2)
- Heart failure	5 (4)	7 (2.3)
- Pulmonary hypertension	12 (9.5)	24 (7.9)
- Extramedullary hematopoiesis	16 (12.5)	15 (4.9)
- Gallstone	44 (34.6)	76 (24.8)
- Fracture	4 (3.1)	3 (1)
- Infection	8 (6.3)	31 (10.1)
- Diabetes mellitus	10 (7.9)	5 (1.6)
- Hypothyroid	9 (7.1)	5 (1.6)
- Hypogonadism	14 (11)	7 (2.3)
- Thrombosis	5 (4)	3 (1)
- Leg ulcer	0 (0)	4 (1.3)
Current iron chelation, n (%)		
- No	6 (4.7)	101 (33)
- Deferoxamine	11 (8.6)	31 (10.2)
- Deferiprone	60 (47.3)	123 (40.2)
- Deferasirox	21 (16.5)	12 (3.9)
- Combined deferoxamine and deferiprone	29 (22.9)	39 (12.7)
Side effect of iron chelation		
- Ocular toxicity	0 (0)	1 (0.3)
- Auditory toxicity	2 (1.5)	1 (0.3)
- Elevated liver enzyme	21 (16.5)	87 (28.4)
- Arthralgia	3 (2.3)	9 (2.9)
- Agranulocytosis	0 (0)	3 (1)
- Gastrointestinal disturbance	16 (12.6)	34 (11.1)
- Elevated creatinine	0 (0)	1 (0.3)
Current medications, n (%)		
- Aspirin	66 (52)	107 (34.9)
- Hydroxyurea	6 (4.7)	5 (1.6)
- Anticoagulant therapy	9 (7.1)	5 (1.6)
- Sildenafil	6 (4.7)	8 (2.6)
- Bosentan	1 (0.8)	1 (0.3)
- Hormone therapy	14 (11)	4 (1.3)
- Calcium	48 (37.8)	110 (35.9)
- Vitamin D	34 (26.7)	74 (24.1)
- Vitamin E	11 (8.6)	1 (0.3)
- Zinc sulphate	4 (3.1)	1 (0.3)
- Multivitamin	30 (23.6)	115 (37.5)
Genotype group, n (%)		
- β-thalassemia/Hb E	116 (91.3)	199 (65)
- Homozygous β-thalassemia	11 (8.7)	4 (1.3)
- Hb H disease	-	24 (7.9)
- Hb H disease with Hb CS	-	30 (9.9)
- Hb H disease with Hb Pakse´	-	15 (4.9)
- EABart’s disease*	-	25 (8.2)
- EABart’s disease with Hb CS**	-	3 (1)
- EFBart’s disease with Hb CS***	-	2 (0.6)
- EABart’s disease with Hb Pakse´****	-	2 (0.6)
- EFBart’s disease *****	-	2 (0.6)

Abbreviation: Hb CS = Hemoglobin Constant spring, Hb Pakse´ = Hemoglobin Pakse´

*compound heterozygous Hb H and heterozygous Hb E

**compound heterozygous Hb H with Hb CS and heterozygous Hb E

***compound heterozygous Hb H with Hb CS and homozygous Hb E

****compound heterozygous Hb H with Hb Pakse´ and heterozygous Hb E

***** compound heterozygous Hb H and homozygous Hb E.

**Table 2:** Multivariate analyses of risk factors for disease-related-complications in 127 patients with transfusion-dependent thalassemia (TDT).

Variables	AOR	95% CI of AOR	p-value
**Pulmonary hypertension**			
- Age > 25 years	4.6	1.2–18.3	0.02
- Post splenectomy time > 5 years	10.4	2.2–49.9	0.003
- Hemoglobin < 8 g/dl	1.4	0.3–7.3	0.7
- Serum ferritin > 2500 ng/ml	1.1	0.6–1.8	0.6
**Heart failure**			
- Age > 25 years	1.1	0.2–7.1	0.9
- Post splenectomy time > 5 years	4.3	0.5–40.2	0.2
- Hemoglobin < 8 g/dl	1.0	0.5–2.3	0.8
- Serum ferritin > 2500 ng/ml	NA	NA	NA
**Infections**			
- Age > 25 years	0.1	0.01–0.8	0.03
- Post splenectomy time > 5 years	1.2	0.3–5.6	0.7
- Hemoglobin < 8 g/dl	2.5	0.3–22.5	0.3
- Serum ferritin > 2500 ng/ml	1.0	0.6–1.5	0.7
**Extramedullary hematopoiesis**			
- Age > 25 years	8.1	1.7–37.9	0.008
- Post splenectomy time > 5 years	2.2	0.7–6.9	0.1
- Hemoglobin < 8 g/dl	0.5	0.1–2.1	0.3
- Serum ferritin > 2500 ng/ml	1.0	0.6–1.6	0.8
**Hypothyroidism**			
- Age > 25 years	0.4	0.1–1.9	0.2
- Post splenectomy time > 5 years	3.0	0.7–13.4	0.1
- Hemoglobin < 8 g/dl	0.2	0.06–1.2	0.09
- Serum ferritin > 2500 ng/ml	1.1	0.4–2.7	0.8
**Hypogonadism**			
- Age > 25 years	6.2	1.2–30.6	0.02
- Post splenectomy time > 5 years	4.5	1.2–18.2	0.03
- Hemoglobin < 8 g/dl	1.1	0.2–5.9	0.9
- Serum ferritin > 2500 ng/ml	0.7	0.5–1.1	0.1
**Diabetes mellitus**			
- Age > 25 years	4.6	0.8–26.9	0.08
- Post splenectomy time > 5 years	2.5	0.5–12.7	0.2
- Hemoglobin < 8 g/dl	0.1	0.01–0.4	0.002
- Serum ferritin > 2500 ng/ml	6.2	1.2–30.8	0.02
**Thrombosis**			
- Age > 25 years	3.1	0.3–30.2	0.3
- Post splenectomy time > 5 years	4.2	0.4–39.3	0.2
- Hemoglobin < 8 g/dl	1.1	0.5–2.5	0.8
- Serum ferritin > 2500 ng/ml	NA	NA	NA
**Gallstone**			
- Age > 25 years	2.5	1.1–5.5	0.02
- Post splenectomy time > 5 years	1.2	0.5–2.5	0.6
- Hemoglobin < 8 g/dl	0.8	0.3–1.9	0.6
- Serum ferritin > 2500 ng/ml	0.6	0.3–1.1	0.1

Abbreviation: OR = odds ratio, 95% CI = 95% confidence interval

**Table 3:** Multivariate analyses of risk factors for disease-related-complications in 306 patients with non-transfusion-dependent thalassemia (NTDT).

Variables	AOR	95% CI of AOR	p-value
**Pulmonary hypertension**			
- Age > 25 years	11.0	2.5–48.6	0.002
- Post splenectomy time > 5 years	2.6	1.1–6.9	0.04
- Hemoglobin < 8 g/dl	2.0	0.6–6.8	0.2
- Serum ferritin > 1000 ng/ml	0.9	0.9–1.0	0.2
**Heart failure**			
- Age > 25 years	9.3	0.9–94.1	0.05
- Post splenectomy time > 5 years	7.6	1.1–53.3	0.04
- Hemoglobin < 8 g/dl	0.4	0.06–3.3	0.4
- Serum ferritin > 1000 ng/ml	1.0	0.9–1.0	0.1
**Infections**			
- Age > 25 years	0.9	0.4–2.0	0.8
- Post splenectomy time > 5 years	1.4	0.5–3.7	0.3
- Hemoglobin < 8 g/dl	0.5	0.2–1.2	0.1
- Serum ferritin > 1000 ng/ml	1.1	1.01–1.04	0.04
**Extramedullary hematopoiesis**			
- Age > 25 years	5.3	1.1–24.6	0.03
- Post splenectomy time > 5 years	2.7	0.8–8.6	0.08
- Hemoglobin < 8 g/dl	2.9	0.5–15.5	0.1
- Serum ferritin > 1000 ng/ml	0.9	0.9–1.0	0.1
**Hypothyroidism**			
- Age > 25 years	0.9	0.1–6.1	0.9
- Post splenectomy time > 5 years	1.7	0.1–24.1	0.4
- Hemoglobin < 8 g/dl	0.1	0.01–1.7	0.1
- Serum ferritin > 1000 ng/ml	0.9	0.9–1.0	0.7
**Hypogonadism**			
- Age > 25 years	0.3	0.05–1.8	0.2
- Post splenectomy time > 5 years	9.5	1.4–64.2	0.02
- Hemoglobin < 8 g/dl	0.9	0.1–6.4	0.9
- Serum ferritin > 1000 ng/ml	0.9	0.9–1.0	0.4
**Diabetes mellitus**			
- Age > 25 years	4.1	0.4–38.6	0.2
- Post splenectomy time > 5 years	0.7	0.06–7.7	0.7
- Hemoglobin < 8 g/dl	1.0	0.1–7.5	0.9
- Serum ferritin > 1000 ng/ml	0.9	0.9–1.0	0.5
**Thrombosis**			
- Age > 25 years	1.3	0.1–16.6	0.8
- Post splenectomy time > 5 years	1.2	0.08–19.9	0.8
- Hemoglobin < 8 g/dl	2.4	0.1–42.3	0.5
- Serum ferritin > 1000 ng/ml	0.9	0.9–1.0	0.09
**Leg ulcers**			
- Age > 25 years	2.8	0.2–30.4	0.3
- Post splenectomy time > 5 years	1.2	0.09–17.0	0.8
- Hemoglobin < 8 g/dl	0.7	0.07–7.8	0.8
- Serum ferritin > 1000 ng/ml	0.9	0.9–1.0	0.1
**Gallstones**			
- Age > 25 years	1.1	0.6–1.8	0.7
- Post splenectomy time > 5 years	1.0	0.5–1.9	0.9
- Hemoglobin < 8 g/dl	1.2	0.7–2.2	0.4
- Serum ferritin > 1000 ng/ml	0.9	0.9–1.0	0.4

Abbreviation: AOR = adjusted odds ratio, 95% CI = 95% confidence interval

Disease-related complications were more prevalent among patients with TDT than those patients with NTDT (100% vs. 58.8%). Advanced age, splenectomy, and iron overload were main clinical risk factors for disease-related complications in patients both with TDT and NTDT. Most of the major disease-related complications were commonly found in the second and third decades of life, except for infections which were more prevalent in the first decade of life and the prevalence decreased slightly with age. Severe iron overload started earlier in patients with TDT than patients with NTDT. Severe iron overload started as early in the first decade of life with the peak incidence in the second and third decades of life in patients with TDT. In patients with NTDT, severe iron overload started in the second decade of life and was high again in the fourth decade of life. This finding might be explained by 1) the high transfusion regimen in children with TDT, 2) Some of the patients with TDT who had several thalassemia-related complications might have died after the third decade of life, and 3) the relatively low serum ferritin when compared to liver iron concentrations in those patients with NTDT might not reflect the true severity of iron overload in these patients.

Almost all of the disease-related complications were more prevalent in patients with transfusion-dependent thalassemia (TDT) than the patients with non-transfusion-dependent thalassemia (NTDT). These findings are similar to the previous study in Thailand.[23]

Endocrinopathies that included diabetes mellitus, hypothyroidism and hypogonadism were more common in patients with TDT. An interesting finding is that iron overload is a statistically significant risk factor for diabetes mellitus in patients with TDT (AOR = 6.2, 95%CI (1.2–30.8), p-value = 0.02). Therefore, screening for diabetes mellitus should regularly be performed in TDT patients with iron overload.

The prevalence of infection is high among patients in the first decade of life and the prevalence is slightly decreased in advanced age. This finding might be explained by the poor immunity in younger patients and the immune system improved with age. Infections were more prevalent in patients with NTDT than TDT in this cohort. This finding might be explained by 1) the larger number of patients with NTDT enrolled in this study, and 2) the higher percentage of patients with NTDT who underwent splenectomy than patients with the TDT group (62.4% vs. 44.1%). Iron overload was a significant risk factor for infections in patients with NTDT (AOR = 1.1, 95%CI (1.01–1.04), p-value = 0.04). This finding is similar to the study by Teawtrakul N *et al*. They found that serum ferritin more than 1,000 ng/ml significantly increased the risk of severe bacterial infections in patients with NTDT (OR = 8.3, 95%CI (1.1–66), p-value = 0.04).[24]

An interesting finding is that pulmonary hypertension is more prevalent in those patients with TDT in this cohort. These results may be due to inadequate blood transfusion in patients with TDT. Previous studies have demonstrated that regular blood transfusions might reduce the pulmonary pressure in patients with thalassemia.[25] Advanced age and splenectomy remained the important clinical risk factors for pulmonary hypertension as indicated in the literature in both groups of patients.[15–17]

Extramedullary hematopoiesis is more common in patients with TDT. Advanced age is the significant risk factor in both patients with TDT (AOR = 8.1, 95%CI (1.7–37.9), p-value = 0.008) and patients with NTDT (AOR = 5.3, 95%CI (1.1–24.6), p-value = 0.03). This finding is similar to the previous study by Teawtrakul *et al*. They found that age greater than 25 years and thalassemia are main clinical risk factors for developing paraspinal extramedullary hematopoiesis.[26] It means that extramedullary hematopoiesis is a time-dependent disease-related complication in patients with thalassemia especially in those patients with a history of inadequate blood transfusion therapy. Previous studies have shown that regular blood transfusions could suppress the erythropoietin levels secondary to ineffective erythropoiesis in this patients.[27,28]

Deferiprone is the most common iron chelation treatment in both patients with TDT and NTDT. This might be explained in that deferiprone in Thailand (GPOL1) can be used in all healthcare schemes; Deferasirox is only allowed to be used in patients with government healthcare insurance. The low percentage of patients using deferasirox is caused by the accessibility to the drug.

This study provides for the importance of early clinical screening and management in particular complications. For example, in TDT patients with severe iron overload should be screened for diabetes mellitus. Screening echocardiography should be performed in advanced age patients who have undergone splenectomy.

The study has some limitations. First, this is an overview of the management of patients with thalassemia in Thailand, therefore the management of these patients was based on the local practice guidelines. Second, the diagnosis of iron overload was mainly based on serum ferritin, because the gold standards, MRI T2*, was not available at all centers. Third, the diagnosis of pulmonary hypertension by using RVSP > 36 mmHg may have caused an overestimation of the prevalence of pulmonary hypertension in these patients. A prospective cohort study in the future will demonstrate the clinical risk factors and the overall survival in patients with thalassemia.

## Conclusions

Disease-related complications are more prevalent in patients with TDT than those patients with NTDT. Advanced age and splenectomy are the significant clinical risk factors for most of the disease-related complications in both groups of patients. Severe iron overload started earlier in patients with TDT and was significantly associated with diabetes mellitus in these patients. Early screening and management for specific disease-related complications should be considered in patients with thalassemia according to their clinical risk factors to improve quality of life in patients with thalassemia.

## Supporting information

S1 File Thalassemia data set. (XLSX)
